# Next-Generation Probiotics as Novel Therapeutics for Improving Human Health: Current Trends and Future Perspectives

**DOI:** 10.3390/microorganisms12030430

**Published:** 2024-02-20

**Authors:** Mohamed E. Abouelela, Yosra A. Helmy

**Affiliations:** 1 Department of Pharmaceutical Sciences, College of Pharmacy, University of Kentucky, Lexington, KY 40536, USA; 2 Department of Veterinary Science, Martin-Gatton College of Agriculture, Food and Environment, University of Kentucky, Lexington, KY 40546, USA

**Keywords:** next-generation probiotics, targeted delivery, therapeutic applications

## Abstract

Next-generation probiotics (NGPs) represent an innovative group of beneficial bacteria that are currently undergoing research and development. NGPs are designed not only for conventional use as foods or dietary supplements but are also tailored for pharmaceutical applications. Research indicates that NGPs show therapeutic promise in addressing various chronic ailments. Offering multiple advantages over conventional probiotics, NGPs present opportunities for personalized probiotic therapies, involvement in synthetic biology and gene editing, participation in combination therapies, targeted delivery methods, and application in therapeutic settings. Our review discusses the potential therapeutic effect of the NGPs, covering diverse research trajectories for NGPs, including their identification, characterization, and targeted delivery. Furthermore, this review elucidates the influence of NGPs on critical aspects of human health, specifically, gut health, immune function, and broader health outcomes. Mechanistic insights encompass the production of bioactive compounds, competitive interactions with pathogenic bacteria, the modulation of immune cell activity, and the reinforcement of the gut barrier. What is noteworthy is that the current review points out the prevalent NGP strains and their diverse sources, providing a highlight for the comprehensive framework for understanding their potential applications and their future benefits in the domain of advanced therapeutics.

## 1. Introduction

Probiotics research has a long history that extends back hundreds of years. The term “probiotics” was primarily assigned to describe the microorganisms and substances that contribute to intestinal microbial balance which was further modified several times to the current definition outlined by the joint FAO/WHO group as “living microorganisms that, when administered in sufficient quantities, offer a health benefit” [1]. Initially, scientists focused on the use of probiotics to prevent and treat gastrointestinal infections caused by certain strains of bacteria that could replace harmful bacteria in the gut and prevent the growth of pathogenic microorganisms [2]. Probiotic research has expanded to include the investigation of other health benefits, such as their effect on immune system modulation and the production of bioactive compounds [3,4]. This resulted in the development of probiotic products for a wide range of applications, including oral health, skin health, and the treatment of specific health conditions such as allergies, metabolic disorders, cancer, and inflammatory bowel disease [5,6]. The field of probiotics research continued to evolve in the twentieth century, with the introduction of new technologies such as genetic sequencing and bioinformatics. As a result, scientists were able to discover and investigate novel probiotic strains [7]. Recently, the field has seen the emergence of next-generation probiotics (NGPs) and live biotherapeutic products (LBPs) with enhanced properties, such as those produced via synthetic biology [8,9].

Next-generation probiotics are live microorganisms identified based on comparative microbiota analyses that, when administered in adequate amounts, confer a health benefit on the host [10,11]. LBPs, on the other hand, are biological products that contain live organisms, applicable to the prevention, treatment, or cure of a disease or condition in humans, and are not vaccines [10]. These products are derived from next-generation sequencing and bioinformatics analysis, and they represent microbial genera and species that have never before been used in the food or pharmaceutical industries [12,13]. NGPs are different from traditional ones in their identification via bioinformatics and/or next-generation sequencing studies, evaluation of their safety and toxicological studies in agreement with novel food regulations, well-defined mode of action, a wide spectrum of microbial genera and species, targeting specific diseases, and potential use as biotherapeutics (Table 1) [12,14]. NGPs are considered potential tools for reducing oxidative stress, modulating inflammatory pathways, and preventing neurodegenerative and viral diseases by regulating the gut microbiome [15].

Probiotics can be provided as dietary supplements, drugs, live biotherapeutic agents, medical food, functional food, and natural products [14]. Probiotics are typically marketed, after receiving FDA institutional approval, as dietary food supplements after its evaluation to ensure the safety [16]. However, the regulatory process for the development and approval of NGPs, including those made through recombinant or genetically engineered methods, is still under investigation. NGPs must go through extensive research and testing through carefully designed preclinical and clinical trials to make sure they are safe for human use and effective in providing the desired health benefits [8]. Despite significant advances in probiotics research, discoveries and technologies emerge over time, and the potential health benefits of probiotics are still being studied as the field evolves. In the current review, we explore the recent advances in the identification, characterization, production challenges, and delivery of NGPs as well as their role and mechanisms for the treatment and control of human diseases as an emerging field for therapeutics development.

## 2. Review Strategy and Literature Survey

To gather articles for our review paper, we conducted searches on the Scopus, PUBMED, and Google Scholar databases. We specifically looked for published English-language articles. Our search terms included “next generation probiotics”, “Characterization of next generation probiotics”, “Production Challenges for next generation probiotics”, “Delivery of the Next-Generation Probiotics”, “Mechanisms of Action of NGPs”, the name of the selected diseases together with “Next-Generation Probiotics”, the diseases together with different genera of the most important probiotic microorganisms, and “Synthetic Biology and Genetic Modification of NGPs”. As a result, we were able to gather 208 publications, which were subjected to our further research and description in later parts of the current review.

## 3. Identification and Characterization of New Probiotic Strains

Probiotics are found in traditionally fermented food, human breast milk, human and animal gut, and the intestinal tracts of marine and freshwater fish [17]. Advances in genetic sequencing and bioinformatic analysis have enabled the identification and investigation of new probiotic strains. Dairy and dairy-related products contain probiotics such as lactic acid bacteria (LAB), bifidobacteria, and other microorganisms derived from fermented milk, which have been used for centuries [18,19].

**Figure 1:**
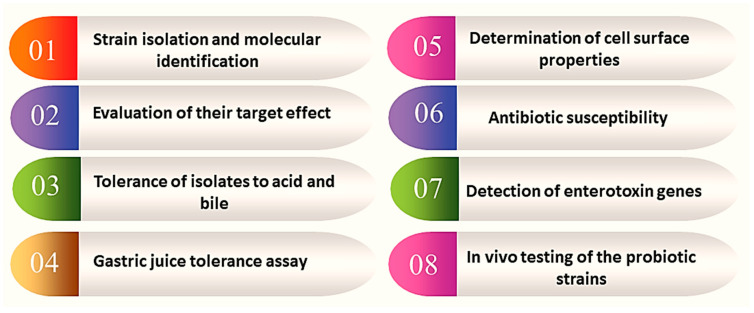
A diagram representing the sequential steps for the characterization of probiotics.

The identification and development of potential probiotics from many ecosystems undergo several steps including isolation and identification, taxonomic classification using genotypic and phenotypic methods, and characterization and evaluation (Figure 1). The analysis of the 16S RNA alone and/or in combination with other methods is used to identify bacterial communities in the gut or ecological sources [17,20]. Polyacrylamide gel electrophoresis (PAGE) with temperature or chemical denaturation, hybridization with fluorescent oligonucleotide probes that target specific 16S, or restriction enzyme digestion (terminal restriction fragment length polymorphism) are common techniques used in combination with 16S RNA analysis [17]. In addition, the 16S to 23S intergenic spacer region has a high degree of sequence and length variation and has been used to distinguish prokaryotic species [21]. This step normally ends with the construction of phylogenetic trees for the selected probiotics strains. The next step is to screen a collection of probiotic strains in vitro for their ability to inhibit the growth of a pathogenic organism or achieve their target effect such as immunomodulatory or disease targeting. Probiotic strains are tested against different pathogenic bacteria using co-culture and antagonistic activity assay techniques to determine their ability to inhibit the pathogen’s growth (Figure 1) [22,23]. 

The immune modulatory assays involve testing the ability of probiotic strains to modulate the immune system by measuring the production of different cytokines and the activation of immune cells [24,25,26]. Strains that can exhibit the desired effect would be considered for further testing. Detailed steps for the development of probiotics were reviewed previously [27,28]. Furthermore, different culture methods are used involving growing probiotic strains in a laboratory setting using different types of media such as MRS (de Man, Rogosa, and Sharpe) and M17. This allows for studying the growth characteristics of probiotic strains, as well as their ability to produce beneficial compounds such as lactic acid and short-chain fatty acids (SCFAs) and modulate the immune system by growing the strains in a simulated gut environment [29]. In addition, simulated gut environments, such as artificial stomach and intestinal fluids, are used as in vitro gut models that involve evaluating the survival, growth, and metabolism of probiotic strains in gut-like conditions and studying the probiotic strains’ behavior in a close approximation of human gut conditions [30]. Furthermore, with the use of organoids, intestinal tissue cultures, or biopsies from humans or animals, the ability to adhere to the intestinal cell epithelium or mucosa is a prerequisite for persistence and colonization. Consequently, it is considered one of the most important selection criteria for newly isolated probiotics, assuring the ability of new candidates to adhere to the human mucosa [31,32]. 

The in vivo testing of the probiotic strains is essential to evaluate their safety and efficacy in reducing the colonization and/or replication of pathogenic bacteria in the gut [25]. This can be carried out by administering the investigated strains to animal models of pathogenic infection and measuring the number of pathogenic bacteria in the gut and their effect on inflammation [33]. In addition, advanced analytical methods such as transcriptomics, proteomics, and metabolomics are used to measure metabolic changes and protein expression in response to probiotic treatment for the identification of new biomarkers of probiotic efficacy and provide a better understanding of the mechanisms of action of probiotics [34]. Recently, the development of high-throughput sequencing, including microbiome analysis methods, allows the analysis of the microbial community in the gut before and after probiotic treatment for evaluating the effects of probiotics on the gut microbiome and identifying new probiotic strains [35].

As a summary, the researchers can identify and isolate NGPs that have the potential to modulate the gut microbiota and improve human health through the following steps: (1) Identify significant microbiota bacteria-host correlations: The first stage is to unravel whether there are significant microbiota bacteria–host correlations in the disease and experimental groups, including growth dynamics, antibiotics sensitivity pattern, and underlying molecular ameliorative mechanisms [36]. (2) Screening and isolating NGPs: next-generation sequencing (NGS) can be used to screen and isolate NGPs. This process involves isolating potential strains with health benefits and using them as candidates for NGPs [12]. (3) Functional validation: rigorous functional validation of the new probiotics is necessary to ensure their safety and effectiveness. This can be achieved through various methods, such as the co-isolation and cultivation of specific bacteria strains [37]. (4) Exploring the mechanism between NGPs and human diseases: understanding the exact mechanism of interaction between NGPs and the host is crucial for finding candidate NGPs for specific diseases. This can be carried out by studying the physiological safety, pathogenicity, drug resistance, and effects on host health and diseases [12]. (5) Reserving microorganism–microorganism interactions to isolate bacteria that can serve as NGP. This can be achieved by plating fecal material from a healthy individual directly on agar plates of specific media, such as Postgate’s medium, and incubating them under anaerobic conditions [37]. And, (6) the in vitro testing of the probiotic strains to evaluate their safety and efficacy in combating pathogenic bacteria in the gut [25].

## 4. Production Challenges for the NGPs

There are several challenges faced in the manufacturing of NGPs. One challenge is the selection and characterization of appropriate probiotic strains. The strains used in probiotics must be suitable for human consumption, have a history of safe use, and possess beneficial properties. However, due to the high diversity of strains and species, it is difficult to select the most appropriate strains for a specific health benefit [38]. Another challenge is the survival of probiotics during storage and gastrointestinal transit. Probiotics must be able to survive the harsh conditions of the stomach and small intestine to reach the gut, where they can exert their beneficial effects [39]. Many probiotic strains are sensitive to changes in pH, temperature, and oxygen levels, which can affect their survivability during storage and transit [40]. The third challenge is the production of probiotics on a commercial scale. Probiotic strains are often grown in laboratory conditions, which can be difficult to replicate on a commercial scale. Additionally, probiotic strains may require specific growth conditions and nutrients that may not be easily available in large quantities [41,42]. Finally, the stability and shelf life of probiotics are also a challenge. The probiotic strains should maintain their viability and functional characteristics during storage and distribution [43,44]. 

Some of the challenges in the production of NGPs include the need for better culturing methodologies, more affordable genome and metagenome sequencing, and more powerful tools to edit and modify bacterial genomes. Additionally, the development of NGPs requires the completion of preclinical safety trials, as well as safety and efficacy trials in humans [7]. Furthermore, the use of genetically modified microorganisms (GMMs) in NGPs raises additional regulatory and consumer acceptance issues. The development of NGPs also involves operational differences, as they are often investigated by laboratories previously engaged in probiotic and microbiome research and have a development trajectory based on the probiotic experience in the laboratory [7]. Moreover, the term NGPs serves a useful purpose in emphasizing that these organisms differ from traditional probiotics in how they are likely to be viewed by regulators and consumers. 

Collaboration among researchers, manufacturers, and regulatory agencies can help to overcome these production challenges. To protect probiotic strains during storage and gastrointestinal transit, for example, we should facilitate the development of new specialized delivery systems such as encapsulation or microencapsulation [45]. Furthermore, optimizing manufacturing processes can aid in improving product consistency, stability, and quality control [46]. This may entail fine-tuning production protocols, incorporating quality control measures, and putting in place monitoring systems to ensure batch-to-batch consistency. 

The regulations for NGPs in the US and Europe are still evolving and lack harmonization. In Europe, the regulatory framework for probiotics has been criticized for failing consumers and the industry by restraining key information on scientifically backed products and impeding market growth. The situation in the EU is not currently harmonized, with different member states taking different approaches to the regulation of probiotics [47]. In the US, the registration of probiotics is regulated by the Federal Food, Drug, and Cosmetics Act (FFDCA) [48]. The regulatory process for probiotic products as live biotherapeutics to treat, cure, or prevent diseases remains challenging globally due to the multifactorial modes of action and strict criteria related to clinical safety, efficacy, and quality. To register a probiotic as a drug in Russia, it must comply with the stipulations outlined in Federal Law No. 61-FZ on Medicine Circulation. The regulation of these medicinal products falls under the purview of the Ministry of Healthcare of the Russian Federation, as per Order No. 403n, issued on July 11, 2017 [48]. This order approves guidelines governing the dispensation of drugs for medical use, including immunobiological drugs, by pharmacies and licensed private entrepreneurs. The regulations for NGPs are still in development, and there is a need for clear guidelines for identifying probiotic strains, their safety evaluation, and regulation [49]. Overall, the production of NGPs involves a range of scientific, regulatory, and consumer acceptance challenges, which need to be addressed for the successful development and commercialization of these novel microbial therapeutics.

## 5. Delivery of the NGPs to the Host

There are several challenges facing probiotics to maintain their viability when they pass through the gut, including variable pH values and high levels of bile salt. Therefore, the appropriate protection of probiotics is essential for developing probiotics as functional food products. Scientists have developed several technologies to make probiotics more resistant to external stress and improve their ability to survive in the gut and enhance colonization. This involves making changes through modifications of the food matrix and engineering techniques during the manufacturing process [50]. The encapsulation of probiotics formulations can protect these probiotic live microorganisms, enhance their stability, and provide advantages of targeted delivery [51,52]. To optimize probiotic encapsulation methods, microbial stability, functionality, safety, efficacy, and targeting ability must be determined both before and after the encapsulation process [53]. Several factors, including the processing temperature, pH of the encapsulating or matrix material, oxygen levels (particularly for certain probiotics), the existence of competing bacteria, and the toxicity of metabolites, have an impact on the viability of probiotics [54]. 

The encapsulation process of probiotic bacteria inside biomaterials and coating agents, such as chitosan, alginate, polysaccharide, alginate–starch, cellulose dietary fibers, rennet-gelled protein, and whey protein gel [55,56,57], provides the protection and stabilization of probiotics during processing, storage, and delivery by enhancing their stress resistance and enabling targeted delivery [58]. There are several formulations for colon-targeted delivery such as polymeric/lipid-coated, pH-controlled, magnetic/enzyme-triggered, and ligand–receptor-based delivery systems [53]. Combining probiotics with prebiotics called “synbiotics” improves the survivability of probiotics and stimulates the growth of specific native strains in the GI tract [59]. 

The selection of the coating agent depends on its functionality, film-forming ability, stability, solubility, digestibility, and releasing properties [60]. To achieve the desired properties, a combination of wall materials or the addition of emulsifiers/filling agents can be used [51]. The most prevalent encapsulation methods for probiotics primarily prioritize their survival rather than preserving their function or distinctive characteristics. It is crucial to recognize that the encapsulation and drying process could influence the surface properties of the cells and their functionality [61]. Researchers and the industry have explored different methods of encapsulation to protect and stabilize probiotics during processing, storage, and delivery. The emerging microencapsulation techniques including spray drying, emulsion technique, freeze-drying, the extrusion technique, spray freeze-drying, electrohydrodynamic processes, reactance window drying, 3D printing, and microfluidics are considered effective encapsulation approaches that support the NGPs’ delivery efficiency [53,59].

## 6. Mechanisms of Action of NGPs 

**Figure 2:**
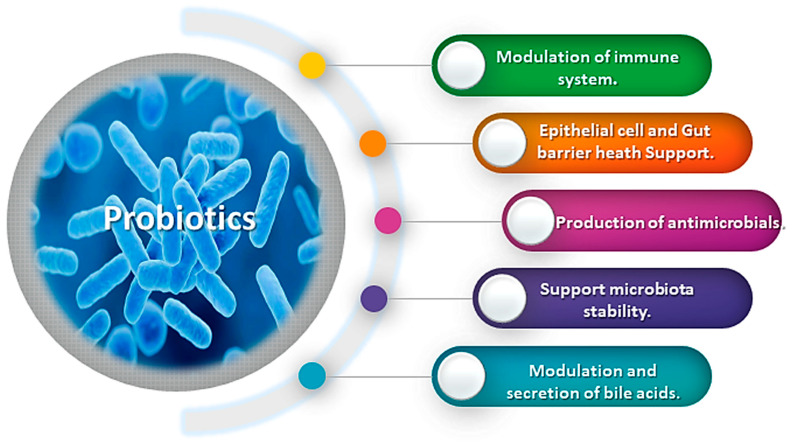
Mechanisms of NGPs health modulation.

Probiotics can have a variety of effects on the host (Figure 2), including changes in the gut microbiome, modulation of the immune system, and production of bioactive compounds. The mechanisms of action of the NGPs on gut health, immune function, and other health outcomes can vary depending on the specific strain or formulation [53,62]. However, probiotics have a wide range of beneficial effects on gut health and the immune system. They produce beneficial compounds such as short-chain fatty acids (SCFAs) and lactic acid and increase the production of antimicrobial peptides like lactobin A, curvacin A, enterocin and pediocin in the gut. These peptides play a crucial role in the defense against pathogenic microorganisms and help maintain a healthy gut microbiota, kill pathogenic bacteria and boost the immune system [63]. NGPs can also modulate the production and secretion of bile acids, which are not only essential for the digestion and absorption of fats but also act as signaling molecules in various metabolic processes. The dysregulation of bile acid metabolism has been implicated in several metabolic and inflammatory disorders, and the ability of NGPs to modulate this process holds therapeutic potential [64]. They also compete with pathogenic bacteria for nutrients and space in the gut, thereby improving mucus production and strengthening the intestinal epithelial barrier which result in reducing the growth of harmful bacteria and promoting a healthy gut microbiome. Probiotics can also modulate the immune system by influencing the activity of immune cells (T-cells and B-cells) and regulating the inflammatory response. Additionally, they can help strengthen the gut barrier, preventing harmful substances from entering the body and enhancing the absorption of nutrients, particularly in the small intestine [53,62,65]. According to recent reports, the *Faecalibacterium prausnitzii* showed ability to induce the anti-inflammatory cytokine IL-10 in peripheral blood mononuclear cells which is likely due in part to their production of short-chain fatty acids or butyrate [66]. Extracellular vehicles (EVs) produced by probiotics have garnered significant attention due to their potential biological activity and future applications. These vesicles serve as a means of transporting various macromolecules, including lipids, polysaccharides, proteins, and nucleic acids, and have been associated with health-inducing properties [67]. Previous research has demonstrated that EVs play a role in modulating inflammatory responses and immune function. For instance, EVs produced by the probiotic *Propionibacterium freudenreichii* CIRM-BIA 129 have been shown to mitigate inflammation by modulating the NF-κB pathway and influencing the activity of immune cells [68].

## 7. Role of the NGPs in the Treatment and Control of Human Diseases

### 7.1. NGPs and Cancer

NGPs have been studied for their potential use in treating various types of cancer, including colorectal cancer (CRC), gastric cancer, hepatocellular carcinoma (HCC), and cervical cancer. However, it is important to note that more research is needed to establish the efficacy of probiotics in treating these cancers [69]. Carcinogenic and inflammatory stimuli can alter the gut microbiota, leading to increased tumor susceptibility. Studies have shown that the mechanisms of probiotic action against colorectal cancer may include the promotion of epithelial repair and barrier function, interference with tumorigenesis inflammatory pathways, apoptosis induction, upregulation of cytokines, production of immunomodulatory metabolites such as SCFAs, acetate, and propionate, biofilm production inhibition and selective exclusion of harmful and tumor-causing microorganisms [70]. In addition, the administration of histamine-producing gut microbes *Lactobacillus reuteri* can reduce inflammation and colon tumor formation [71]. Lower intracolonic pH levels induced by *Lactobacilli* were also seen to inhibit the activity of putrefactive carcinogenic enzymes [72]. Other trial studies on *L. acidophilus*, *L. salivarius*, *L. plantarum*, *L. rhamnosus*, *L. kefiri*, *L. casei*, *L. delbrueckii*, *Bifidobacterium infantis*, *B. breve*, *B. longum*, and *Streptococcus thermophilus* showed promising results in cancer prevention and early-stage colon cancer in animal models [70].

Antiproliferative or proapoptotic effects of probiotics on human cancer cells have been investigated. For example, the *L. rhamnosus* strain GG showed an inhibition effect on both human gastric and colonic cancer cells, while *B. adolescentis* SPM0212, *Bacillus polyfermenticus*, and *L. acidophilus* 606, as well as *B. animalis* subsp. *Lactis,* inhibited the proliferation of HT-29, Caco-2 and SW 480 colon cancer cells [69]. Furthermore, animal models were also used to test the anticancer effects of probiotics, specifically in rats and mice. Colon cancers were induced in these animals using carcinogens such as 1,2-dimethylhydrazine or azoxymethane. Treatment with strains of probiotics such as *L. plantarum*, *L. fermentum* or a combination of *L. acidophilus* and *B. bifidum*, *L. rhamnosus* and *B. lactis*, as well as a combination of *L. acidophilus* and *L. helveticus* was found to significantly inhibit and decrease the incidence of colonic cancer development in rats and mice that had been injected with carcinogens such as 1,2-dimethylhydrazine [69].

Recent studies have identified several potential NGPs for various health benefits, such as *Prevotella copri*, *Christensenella minuta*, *Parabacteroides goldsteinii*, *Akkermansia muciniphila*, *B. thetaiotaomicron*, *F. prausnitzii*, and *B. fragilis* [73]. Among these, *A. muciniphila* has potency in cancer immunotherapy and is enriched in the microbiota of patients who respond well to anti-PD1 blockade therapy by increasing the number of cytotoxic T cells and regulatory T (T_reg_) cells [74,75]. *Butyricicoccus pullicaecorum* has been found to prevent necrotic enteritis and is safe in human trials [76]. It also shows anticancer effects due to its high production of butyrate, which inhibits CRC cell growth and upregulates tumor suppressor genes SLC5A8 and GPR43 [77]. In addition, *L. plantarum* showed positive potential in inducing apoptosis in cancer cells by decreasing the expression of AKT and increasing the expression of PTEN genes, inhibiting the viability of human oral and gastric cancer cells [78,79]. Recent studies suggested that *L. plantarum* XB7 may play a significant role in the prevention of *Helicobacter pylori*-associated gastric cancer via apoptosis induction and the suppression of IL-8 production and IL-8 mRNA expression in *H. pylori*-induced AGS cells without inhibiting *H. pylori* growth [77,79]. In vitro effect of probiotics against breast cancer MDA-MB-231 cells showed the potential effect of *L. plantarum* through apoptosis mediated by the downregulation of the NFκB pathway. In addition, *L. crispatus* and *L. acidophilus* revealed promising effects in an MTT assay against the same cells via the decrease in transcriptional activity of four different cancer–testis antigens [80]. Furthermore, *L. plantarum*, *L. acidophilus*, *L. paracasei*, *L. casei*, *B. infantis*, *L. paracasei*, and *B. bifidum* were observed to reduce breast cancer cell growth [81]. A case study has suggested that the lower incidence of breast cancer in Japanese women is linked to the daily oral administration of *L. casei*. This study suggested that long-term exposure was required to create a chemo-preventive effect of probiotics on cancer growth [82]. Wan et al. also claimed that *L. crispatus* can significantly inhibit the proliferation, induce the apoptosis, and inhibit the cell migration of the cervical precancerous cell line Ect1/E6E7 in a time-dependent manner via multiple mechanisms [83].

The potential of *Salmonella* sp. for cancer therapy was highlighted by its ability to localize and proliferate inside tumor microenvironments and often suppress tumor growth [84]. *Salmonella* can serve as a unique tool in cancer treatment by delivering toxins that specifically trigger cell death in cancer cells. Additionally, they can be employed for cancer-specific immunotherapy by delivering tumor antigens and exposing the tumor environment to the immune system. Another application involves using *Salmonella* to deliver enzymes that convert prodrugs specifically against cancer. While the positive outcomes of *Salmonella*-based cancer treatments are in the early stages, they have laid the groundwork for combining these approaches with traditional chemotherapy, radiotherapy, and surgery. This combined strategy holds promise for effectively tackling multi-drug-resistant and advanced-stage cancers [84]. Additionally, *B. pullicaecorum* could produce butyrate, a short-chain fatty acid with a variety of gastrointestinal health benefits. *B. pullicaecorum* has been shown to be safe in in vitro studies, animal models, and human intervention trials, paving the way for its further development as a NGP [76,85]. This butyrate-producing bacterium has shown promise in attenuating gut inflammation by coupling with a cell-surface G-protein-solute carrier family 5 member 8 (SLC5A8), preventing necrotic enteritis, reducing pathogen abundance, and regulating short-chain fatty acid transporters and receptors to reduce the progression of colorectal cancer [85]. In conclusion, the potential of NGPs in treating various types of cancer, such as colorectal cancer, gastric cancer, hepatocellular carcinoma, and cervical cancer, has been explored in numerous studies. However, more research is needed to evaluate the efficacy of probiotics in treating these cancers. The mechanisms of probiotic action against cancer may include promoting epithelial repair and barrier function, interfering with tumorigenesis in inflammatory pathways, inducing apoptosis, upregulating cytokines, producing immunomodulatory metabolites, inhibiting biofilm production, and selectively excluding harmful and tumor-causing microorganisms.

### 7.2. NGPs and Gastrointestinal Disorders

Gastrointestinal (GI) disorders are conditions that affect the digestive system. GI disorders include gastroesophageal reflux disease (GERD), irritable bowel syndrome (IBS), inflammatory bowel disease (IBD), ulcerative colitis, Crohn’s disease, and diarrhea [86]. Treatment for GI disorders depends on the specific condition but may include lifestyle changes and medication in severe cases or surgery [87].

**Figure 3:**
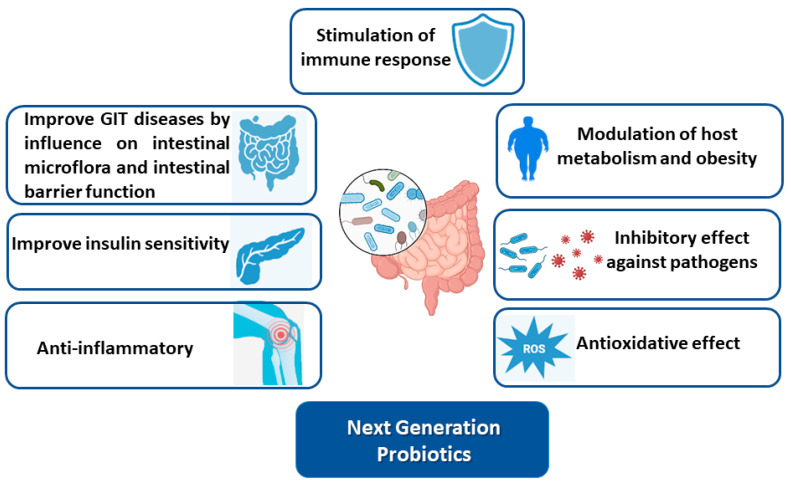
Health benefits of next-generation probiotics.

NGPs can specifically target GI disorders and help to restore the balance of bacteria in the gut, which can improve digestive health and help to prevent or treat various GI disorders [59]. The mechanisms of action of probiotics in gastrointestinal disorders are not fully understood, but several mechanisms have been proposed based on current research (Figure 3). Some of the most commonly discussed mechanisms of action of probiotics in GI disorders are their competition with harmful bacteria for nutrients and space in the gut, helping to reduce the growth and activity of harmful bacteria, which can improve gut health and reduce symptoms of various GI disorders [88]. The stimulation of the immune system to reduce inflammation in the gut is another mechanism, particularly in IBD and other conditions that involve gut inflammation [88]. The modulation of gut motility can improve symptoms of constipation and diarrhea. The production of short-chain fatty acids and the improvement of gut permeability can help to reduce symptoms of irritable bowel syndrome (IBS) and other conditions that involve gut dysfunction [89].

Some of the most commonly used NGPs include *B. pseudopodium*, *L. rhamnosus*, *L. acidophilus*, and *L. lactobacillus* which were reported to treat GI disorders by modulating the serotonergic system in IBS [89]. Martin et al. found that *F. prausnitzii* can reduce intestinal permeability, colonic serotonin, and cytokine levels with low-grade inflammation and intestinal dysfunction induced by dinitro-benzene sulfonic acid-induced low-grade inflammation and intestinal dysfunction in a mouse model [90]. Additionally, *B. bifidum* has been shown to improve symptoms of IBS symptoms such as abdominal pain and bloating, as well as reduce the risk of developing antibiotics-associated diarrhea [91]. A clinical double-blind, placebo-controlled study investigated the efficacy of *B. bifidum* MIMBb75 in treating IBS and showed a significant reduction in overall IBS symptoms such as pain/discomfort, distension/bloating, urgency, and digestive disorder in 47% of the patients who received the *Bifidobacteria* treatment [92]. Furthermore, *B. lactis* has been found to improve gut motility, which can reduce symptoms of constipation. It may also have a positive effect on the immune system, which can help to prevent infections that can cause GI symptoms. Fermented milk containing *B. lactis* CNCM I-2494 improved IBS symptoms in constipated IBS patients [93]. In rats subjected to stress conditions, the symptoms of IBS tend to worsen due to changes in the gut, including increased sensitivity and permeability due to epithelial cell contraction. To investigate whether treatment with *B. lactis* could mitigate these effects, the researchers conducted a study in which rats were given the probiotic. The results showed that *B. lactis* was able to reduce gut hypersensitivity and prevent disruption of the colonic barrier caused by acute stress in rats [93].

Furthermore, *L. acidophilus* has been proven to be beneficial in improving symptoms associated with IBS and IBD. A controlled trial involving adults with IBS, where *L. acidophilus* DDS-1 and *B. lactis* UABla-12 were tested, demonstrated a notable reduction in the severity of abdominal pain and overall symptom improvement compared to a placebo. Those who supplemented with *L. acidophilus* DDS-1 and *B. lactis* UABla-12 also witnessed a normalization of stool patterns and experienced enhanced relief from abdominal pain throughout the intervention period [94]. Other probiotic strains such as *L. rhamnosus*, *L. plantarum*, and *L. reuteri* were found to be effective in reducing inflammation in the gut, which can improve symptoms of inflammatory bowel disease, improve gut permeability and reduce the risk of developing diarrhea [95,96,97]. *S. thermophilus* also improves gut function and alleviates IBS symptoms, as well as lactose digestion, and lowers the risk of developing lactose intolerance [98]. *Blautia producta* D4 was also reported to significantly improve the condition of mice experiencing colitis [99,100]. This improvement was reflected in reduced body weight loss and a decrease in pro-inflammatory cytokines, such as interleukin-6 (IL-6), tumor necrosis factor-α (TNF-α), and interleukin-1β (IL-1β). Moreover, *B. producta* D4 also played a role in mitigating oxidative stress, as evidenced by lower levels of myeloperoxidase (MPO) activity, along with increased activities of antioxidant enzymes like superoxide dismutase (SOD) and glutathione peroxidase (GSH-Px) [99]. Additionally, the level of malondialdehyde (MDA), an indicator of oxidative stress, was reduced. Mao et al. suggests that the oral administration of *B. producta* D4 effectively alleviates DSS-induced colitis by addressing multiple factors. This includes suppressing inflammatory responses, maintaining the integrity of the intestinal barrier, inhibiting the TLR4/NF-κB pathway, and restoring a balance in the intestinal microbiota [99]. In summary, there is high potential for NGPs to treat GI disorders such as GERD, IBS, IBD, ulcerative colitis, Crohn’s disease, and diarrhea. They modulate their function through outcompeting harmful bacteria, boosting the immune system, regulating gut motility, producing short-chain fatty acids, and enhancing gut permeability. However, further research is essential to validate the efficacy of NGPs in managing these disorders.

### 7.3. NGPs and Cardiovascular Diseases

Recent studies have illuminated the pivotal role of gut microbiota modulation in cardiovascular health, opening avenues for the exploration of NGPs as potential tools for the prevention and treatment of cardiovascular diseases [101]. The profound influence of gut microbiota on cardiovascular health is evidenced by its impact on inflammation, insulin sensitivity, and cholesterol levels [101]. Through targeted modulation, NGPs hold promise in maintaining a balanced gut microbiota, offering a compelling avenue for the prevention and treatment of cardiovascular diseases. It was reported that *P. copri* and *C. minuta* stand out for their capacity to regulate insulin resistance, a key factor in cardiovascular diseases [36]. Additionally, Eggerthellaceae bacteria, including *Eggerthella lenta*, which transforms ellagitannins from specific foods into compounds with anti-carcinogenic and cardioprotective effects, contribute to the diverse array of NGPs identified for cardiovascular health [102,103]. *B. thetaiotaomicron* has also been recognized for its role in reversing obesity and insulin resistance, underscoring the potential impact of NGPs on cardiovascular well-being [77]. Nevertheless, comprehensive research efforts are imperative to fully elucidate the mechanisms of action and optimize the therapeutic applications of these NGPs in the context of cardiovascular health [101]. The emerging insights into the significant impact of gut microbiota modulation on cardiovascular health underscore the potential of NGPs as promising tools for the prevention and treatment of cardiovascular diseases. The targeted modulation offered by NGPs, with specific strains like *P. copri*, *C. minuta*, Eggerthellaceae bacteria, and *B. thetaiotaomicron*, holds promise in addressing key factors such as inflammation, insulin resistance, and cholesterol levels.

### 7.4. NGPs and Metabolic Diseases

Metabolic diseases, such as obesity, type 2 diabetes, and non-alcoholic fatty liver disease (NAFLD) are growing health problems worldwide. These conditions are caused by a combination of factors such as genetics, lifestyle, and diet [104]. The gut microbiome is responsible for the digestion and absorption of nutrients, the production of hormones and neurotransmitters, and the regulation of the immune system. An imbalance in the gut microbiome, referred to as gut dysbiosis, has been linked to the development of metabolic diseases [105]. NGPs have been proposed as a promising strategy for the treatment of metabolic diseases [106]. One of the most well-studied NGP in the treatment of metabolic disease is *L. rhamnosus* GG (LGG) which has been shown to improve insulin sensitivity, reduce body weight, and improve lipid metabolism [107]. Sanchez et al. studied its effect on obese individuals with type 2 diabetes, and supplementation with LGG for 12 weeks was shown to improve insulin sensitivity and reduce body weight compared to a placebo group [108]. Another study found that LGG supplementation in obese individuals with NAFLD resulted in a significant reduction in liver fat and an improvement in liver function in mice [109]. Another promising NGP in the treatment of metabolic disease is *B. lactis* which has been shown to improve insulin sensitivity, reduce hemoglobin A1c levels, reduce body weight, and improve lipid metabolism [110]. The use of *B. animalis* subsp. *lactis* BB-12 has been shown to positively impact the gut microbiota in humans. This strain of probiotics has been found to protect the community structure of the gut microbiota and combat obesity. This is achieved by promoting the growth of beneficial bacteria, such as *Prevotella* sp., and reducing the growth of harmful bacteria, such as *Clostridium* sp., *Blautia* sp., and *Bacteroides* sp. As a result, the transition from a healthy state to an obese state was suppressed [111]. Additionally, *B. longum* has been shown to improve body weight and glucose metabolism, as well as reduce inflammation in people with metabolic diseases. While another study revealed that *B. longum* 070103 fermented milk (BLFM) significantly reduces the content of 3-indoxyl sulfate, which is linked to intestinal barrier damage. Furthermore, BLFM treatment improved BW, glucose tolerance, insulin resistance, and hepatic steatosis in mice [112].

Kim et al. suggested a link between *B. longum* and *B. bifidum* abundance and increased visceral adipose tissue (VAT), body mass index (BMI), blood triglycerides (TG), and fatty liver. The carbohydrate and nucleoside metabolic processes of these *Bifidobacterium* strains can protect against diet-induced obesity by improving bile acid signaling, resulting in less body weight gain, improved hepatic steatosis, and improved glucose control. These strains also protect germ-free mice from diet-induced obesity by manipulating the intestinal sterol biosynthetic processes [113]. A study conducted by Hao et al. showed that a daily dose of 10^9^ CFU of *B. longum* significantly decreased fasting blood glucose and improved insulin resistance in diabetic mice. *B. longum* BL21 strain also boosted anti-oxidation, increased hepatic glycogen, and reduced gene expression of G6Pase and PEPCK. *B. longum* also improved endotoxemia-related inflammation and intestinal barrier function and regulated gut flora (*Akkermansia* sp., *Prevotella* sp., *Bacteroides* sp., *Alistipes* sp., *Mucispirillum* sp., and *Odoribacter* sp.). Thus, *B. longum* may be a potential functional food for T2DM amelioration due to the regulation of glucose metabolism and gut microbiota modulation [114]. *L. fermentum*, *L. plantarum*, *L. paracasei*, and *L. rhamnosus* were shown to have a positive effect on glucose metabolism reducing body weight, improving insulin sensitivity, and decreasing blood lipid levels in obese individuals [115,116,117,118,119,120].

*Blautia wexlerae* has emerged as a promising candidate for treating metabolic diseases, particularly obesity and type 2 diabetes [121]. Through animal experiments, it has been observed that the oral administration of *B. wexlerae* to mice significantly reduces obesity and diabetes induced by a high-fat diet [121]. The unique metabolic characteristics of *B. wexlerae* play a key role in delivering these beneficial effects for controlling obesity and type 2 diabetes [121]. One notable aspect is the significant acetate production by *B. wexlerae*, a factor linked to metabolic changes and anti-inflammatory effects. This contributes to the overall reduction in obesity and diabetes [121]. The positive impacts of *B. wexlerae* are associated with the generation of S-adenosylmethionine, acetylcholine, and l-ornithine, as well as its involvement in carbohydrate metabolism. This leads to the buildup of amylopectin and the generation of succinate, lactate, and acetate. As a result, *B. wexlerae* brings about changes in the gut environment, influencing both the bacterial composition and the production of short-chain fatty acids in the gut microbiota [121,122]. Recent research indicates that *C. minuta* plays a crucial role in averting obesity. Studies have revealed that in diet-induced obesity (DIO) mouse models, *C. minuta* effectively curbs weight gain and oversees key metabolic indicators such as glycemia and leptin [123,124]. The strain *C. minuta* DSM33407 has emerged as a promising biotherapeutic candidate for addressing obesity and related metabolic disorders. It has demonstrated the ability to counteract body weight increase, diet-induced hyperglycemia, and leptin levels in the body [123]. Furthermore, *C. minuta* is linked to the modulation of gut microbiota, the generation of short-chain fatty acids, and the regulation of lipid metabolism. These findings underscore its potential as a biotherapeutic agent for tackling obesity and associated metabolic ailments, showcasing its multifaceted impact on various aspects of metabolic health [125].

*Parabacteroides* spp., including *P. goldsteinii*, have emerged as promising candidates for NGPs due to their capacity to regulate insulin resistance and reverse obesity. Studies highlight that *P. distasonis* mitigates insulin resistance through the activation of intestinal GPR109a [126]. Two strains of *P. distasonis* (*P. distasonis* AS93 and PF-BaE11) have demonstrated the ability to reduce obesity and associated disorders, with observed benefits linked to decreased inflammation in adipose tissue [127]. Supplementation with *P. distasonis* has been shown to modify the *Actinomycetota*, *Bacillota*, *and Bacteroidota* taxa in the gut microbiota of mice [127]. Additionally, *P. distasonis* has been associated with improvements in adiposity, glycemia, insulin resistance, and fatty liver indices [128]. The alleviation of obesity and metabolic dysfunctions by *P. distasonis* has been attributed to its production of succinate and secondary bile acids [129]. Together, NGPs show promising potential in the treatment of metabolic diseases such as obesity, type 2 diabetes, and NAFLD. Various strains of NGPs, including *L. rhamnosus* GG, *B. lactis* Bb-12, *B. animalis* subsp. lactis BB-12, *B. longum*, *B. wexlerae*, *C. minuta*, and *Parabacteroides* spp., have been found to improve insulin sensitivity, reduce body weight, and improve lipid metabolism. These probiotics have demonstrated beneficial effects on glucose metabolism, inflammation, and gut microbiota modulation, making them potential candidates for the development of novel therapies for metabolic diseases. However, further research and clinical studies are needed to fully understand their mechanisms of action and to establish their efficacy and safety for routine use in the treatment of these conditions.

### 7.5. NGPs and Neuropsychological Disorders

Probiotics have been traditionally known for their role in maintaining gut health, but recent research has shown that they can also have a potential role in treating various neurological and psychiatric disorders [130]. The gut–brain signaling mechanisms involving the microbiota are currently being studied and understood. These may involve changes in the microbial composition, immune system activation, and signaling through the vagus nerve, as well as changes in tryptophan metabolism and the production of specific neuro-active metabolites by the bacteria. Furthermore, the bacteria also provide various neuroprotective benefits, such as inhibiting beta-amyloid fibril formation and having antioxidant properties, through the same pathways [131]. Tillmann, et al. reported that probiotics influenced two metabolic pathways in the host. They reduced methyl group flow by using betaine and increased liver SAM while decreasing plasma dopamine and norepinephrine [132]. These methylation and catecholamine pathway changes are known to be involved in a variety of diseases, implying that probiotics may play a role in their treatment. It was reported that *L. helveticus* R0052 and *B. longum* R0175 resulted in significant improvement in symptoms of depression in individuals with major depressive disorder (MDD) [132,133]. This study showed that the administration of *L. helveticus* R0052 and *B. longum* R0175 increased liver concentrations of SAM in depressed rats and lowered plasma dopamine levels in a dose-dependent manner. These results provide novel evidence for the influence of probiotics on two biochemical pathways involved in mood disorders [132]. Additionally, *L. rhamnosus* JB-1 was found to reduce depressive-like behaviors in normal and chronic-stressed mice, as well as in postpartum women and obese individuals. Its antidepressant effect is thought to be due to signaling to the brain via the neural route, which may influence the central GABAergic system and HPA axis [130]. A mixed-species probiotic that included *L. casei* was also found to reduce depression and depressive-like symptoms in MDD patients and healthy individuals [134]. *F. prausnitzii* was also reported to have anti-depressive and anxiolytic effects in chronic-stressed mice, and low populations of *F. prausnitzii* were found to be correlated with the disease severity of MDD and bipolar depression [130]. Chronic administration of a probiotic mixture of *L. helveticus*, *B. longum*, *L. lactis*, and *S. thermophilus* was found to affect anxiety- and depressive-like behaviors in rats, possibly due to changes in certain metabolites and brain monoamines [135].

A study conducted by Gu et al. evaluated the effectiveness of *L. casei* intervention on mental disorders in rats using a depression-like behavior model induced by chronic unpredictable mild stress. The results showed that *L. casei* improved depression-like behavior, changed the gut microbiota structure, and reversed changes in protein expression and the activation of signaling pathways. These findings suggest that *L. casei* may have protective effects against depression in rats and be associated with changes in the gut microbiota and BDNF-TrkB signaling [136]. According to a study conducted by Liu et al. in Taiwan, children with autism receiving *L. plantarum* PS128 showed improved opposition and defensive behaviors, especially in younger children. This suggests that this strain of probiotics may help with autism symptoms such as disruptive and rule-breaking behaviors, as well as hyperactivity. *L. plantarum* PS128 was found to be more effective in younger children, highlighting the importance of early intervention [137]. A few studies on the relation between attention-deficit/hyperactivity disorder (ADHD) and probiotics have suggested that probiotics may have a role in improving symptoms of ADHD, particularly in terms of reducing impulsivity and inattention. Preliminary evidence suggests that *L. rhamnosus* GG and *B. animalis* may have a role in reducing symptoms of ADHD by improving gut health and reducing inflammation [138]. Additionally, some studies have suggested that probiotics may have a role in reducing the risk of Alzheimer’s disease by reducing inflammation and oxidative stress in the brain [139,140]. *L. paracasei* have also shown a neuroprotective effect by reducing inflammation and oxidative stress in the brain, making it a potential candidate for the treatment of neurological disorders such as Alzheimer’s disease [141,142]. A combination of three probiotics, *L. reuteri*, *L. rhamnosus*, and *B. infantis*, was able to decrease the amount of A plaques in rats with Alzheimer’s disease (AD). The treatment group also had lower levels of MDA (a marker of oxidative stress) and higher levels of SOD (an antioxidant enzyme), suggesting that the probiotics improved overall antioxidant status [139]. In addition, the treatment led to a reduction in two markers of inflammation, IL-1, and TNF-α, in the rat AD model [139,140].

Additionally, probiotic *P. freudenreichii* P.UF1, sourced from human breast milk, is currently being marketed for its potential advantages in supporting immune health and producing vitamin B12 [143]. *P. freudenreichii* stands out by generating significantly higher levels of vitamin B12 in comparison to other identified strains of *P. freudenreichii*. Vitamin B12 is crucial for the proper functioning of the brain, nervous system, and blood production. Additionally, *P. freudenreichii* is known for having a pathway for synthesizing vitamin B12, further highlighting the potential health benefits associated with P.UF1 [143]. *P. freudenreichii* is also beneficial in modulating the gut microbiota, motility, and inflammation [144]. Taken together, NGPs have shown potential in treating various neurological and psychiatric disorders, including depression, anxiety, autism, ADHD, and Alzheimer’s disease. They may influence gut–brain signaling mechanisms, change microbial composition, activate the immune system, and produce neuro-active metabolites. They can also provide neuroprotective benefits, such as inhibiting beta-amyloid fibril formation and having antioxidant properties. 

### 7.6. NGPs and Skin Diseases

The use of NGPs has shown promise in improving skin health and treating various skin conditions. Some studies have found that probiotics, such as *L. acidophilus*, *L. fermentum*, and *B. bifidum*, can help to reduce skin inflammation and improve symptoms of conditions such as acne, atopic dermatitis, and rosacea. Probiotics can also improve the overall balance of the skin microbiome and boost the skin’s natural defenses against harmful pathogens [145,146,147]. Orally administering *L. acidophilus* to rats with wounds resulted in notable improvements in wound contraction and reduced time for complete epithelialization in excision wounds. Additionally, there was an increase in the dry weight of granulomas and cellular infiltration in the granulation tissue, along with a significant rise in collagen content. These findings indicate enhanced wound healing [148].

What is noteworthy is that oral supplementation with *B. breve* B-3 has demonstrated significant inhibitory effects on transepidermal water loss (TEWL), skin dryness, and changes in epidermal thickening in mice exposed to excess UV irradiation. Moreover, *B. breve* B-3 supplementation has shown improvements in the integrity of tight junction structures and basement membranes following UV-induced injury. Additionally, the supplementation has been found to suppress the UV-induced production of IL-1β in the skin [149].

Furthermore, when *Bifidobacterium* and *Lactobacillus* are administered in lyophilized powder form within capsules, they exhibit a suppressive effect on atopic sensitization to common food allergens and contribute to a reduction in the prevalence of atopic eczema in early childhood [149]. According to a clinical trial on the effect of a probiotic mixture containing *B. bifidum*, *B. lactis*, and *L. acidophilus*, it has been found that supplementing before and after birth with this mixture is an effective way to prevent the onset of eczema in infants who are at a high risk of developing allergies during their first year of life [146]. Additionally, bacteriocins produced by *S. salivarius*, *Lactococcus* sp. HY 449, and *L. salivarius* LS03 showed an inhibitory effect on the growth of *Cutibacterium acnes*. Because of its antimicrobial activity, *B. adolescentis* SPM0308 was effective in controlling the growth of both *C. acnes* and *S. aureus.* Similarly, Gueniche et al. reported that *L. paracasei* CNCM I-2116 improved key mechanisms associated with skin barrier function, such as TNF release induced by substance P [150]. The emerging NGPs are starting to reveal new preventive and therapeutic potential in skin health.

### 7.7. NGPs and Infectious Diseases Control

Probiotics as a treatment for infectious diseases is a rapidly expanding field, with a growing body of evidence indicating that certain probiotics can help boost the body’s natural defenses against certain infections. NGPs will almost certainly involve the use of specific strains of beneficial bacteria that have been chosen for their ability to target specific pathogens or modulate the immune system in a way that is beneficial for the treatment of specific infections [88]. Probiotics have been shown in some studies to help prevent or treat infections caused by bacteria such as *E. coli*, *Salmonella* spp., and *S. aureus.* According to other research, probiotics can help boost the immune system and prevent the onset of respiratory and gastrointestinal infections [151].

*L. reuteri* has been shown to have antimicrobial activity against a variety of pathogenic bacteria, including *E. coli*, *S.* Typhimurium, and *H. pylori* [152]. It has also been demonstrated to stimulate the production of antibacterial peptides [153] and enhance the immune response to bacterial infections. *L. reuteri* has been shown to have a positive impact on the gut microbiome and the growth of beneficial bacteria [154]. It has also been shown to have a positive effect on the immune system, enhancing the production of cytokines and increasing the activity of white blood cells [155]. Additionally, *L. rhamnosus* has been shown to enhance the immune response against bacterial infections and to have a protective effect against infections caused by *E. coli* and *Salmonella* [156,157,158]. The *L. acidophilus* strain has had a positive impact on the gut microbiome, reducing the growth of pathogenic bacteria and promoting the growth of beneficial bacteria. It has also been shown to have a positive effect on the immune system, stimulating DCs to release the IL-4 cytokine and increasing the activity of white blood cells [88,159,160]. Other lactobacilli strains such as *L. fermentum* [161], *L. paracasei* [162], *L. casei* [163], *L. helveticus*, *L. crispatus*, and *L. johnsonii* have also been shown to combat activity and reduce the growth of pathogenic bacteria like *Salmonella enterica* subsp. *enterica* and *S. aureus* and enhance the immune system [164]. Another example is *Bacillus coagulans*, which has been shown to have a broad-spectrum antibacterial activity against a range of pathogens, including *S. aureus* and *C. difficile* [165]. It has also been shown to have a positive impact on the gut microbiome, increasing the diversity of beneficial bacteria and reducing the growth of pathogenic bacteria [166].

The *S. thermophilus* strain has been shown to have a positive impact on the gut microbiome, reducing the growth of pathogenic bacteria and promoting the growth of beneficial bacteria. It has also been shown to have a positive effect on the immune system, enhancing the production of cytokines and increasing the activity of white blood cells [167]. *B. lactis*, *B. longum*, and *B. bifidum* probiotic strains have been shown to enhance the immune response to viral infections and to have a protective effect against infections caused by *Salmonella* serotypes and *E. coli* [168,169].

Studies have shown that the gut microbiota such as *Lactobacillus* and *Bifidobacterium* is reduced and that other bacteria such as *Collinsella*, *Streptococcus*, and *Morganella* are more abundant in patients infected with SARS-CoV-2. The most prevalent commensals in a healthy population are *Eubacterium* sp., *F. prausnitzii*, *Roseburia* sp., and *Lachnospiraceae* [170]. These commensals were reduced in COVID-19 patients, while opportunistic pathogens such as *C. hathewayi*, *Actinomyces viscosus*, and *B. nordii* were increased. Due to their ability to regulate the immune system, probiotic strains such as *F. prausnitzii*, *Eubacterium rectale*, and *Bifidobacteria* showed a potential reduction in the individuals affected by COVID-19. Furthermore, investigations utilizing a non-human primate model have revealed that SARS-CoV-2 infection induces changes in both the composition and functionality of the intestinal microbiota [170]. Thus, NGPs could be used as an immunomodulatory complementary treatment for COVID-19 infections.

*Pediococcus pentosaceus* is a promising NGP lactic acid bacteria (LAB) with a good safety profile, flavor enhancement, food preservation, and pathogenic bacteria inhibition effect [171]. *P. pentosaceus* has been highlighted for its probiotic effects, including its antibacterial mechanisms, such as the secretion of bacteriocins or bacteriocin-like substances (BLISs) to damage cell walls or to directly kill pathogenic bacteria, effectively combating bacterial threats [171,172]. A study conducted by Sang-Kyu et al. showed that *P. pentosaceus* possesses potential activity against several bacterial species such as *S. aureus*, *S. epidermidis*, *P. aeruginosa*, *P. Acnes*, *P. putida*, *S. xylosus*, *E. coli*, *B. cereus* and *B. vallismortis* [173]. The use of specific strains of beneficial bacteria, such as *Lactobacillus*, *Bifidobacterium*, and *B. coagulans*, has been shown to target specific pathogens and modulate the immune system, enhancing the body’s natural defenses against infections. Further research is needed to fully understand the mechanisms behind the effectiveness of probiotics and to develop more targeted and effective therapeutics for the infectious diseases.

## 8. Synthetic Biology and Genetic Modification of NGPs for Specific Applications

Recently, NGPs have been considered as advanced forms of probiotics that produce specific metabolites, target specific diseases, or are genetically engineered or modified to enhance their beneficial effects on human health [8]. These probiotics are designed to target specific health conditions and offer improved efficacy and potency compared to traditional probiotics. Modified NGPs are being developed for a variety of targeted applications relying on gene editing technology, such as oral health, skin health, and the management of specific health conditions such as allergies, metabolic disorders, cancer, and inflammatory bowel disease [174]. Existing probiotics are modified using gene editing technology such as zinc-finger nucleases (ZFNs), transcription activator-like effector nucleases (TALEN), clustered regularly spaced short palindromic repeats (CRISPR), CRISPR-associated protein (CRISPR-Cas) and CRISPR-Cas-9 to create the desired new probiotics [174]. These engineering modifications help to directly verify whether the genetic material and its functional roles have been altered as required [174].

Engineered probiotics have been used to target many diseases and health issues in both humans and animals. For example, some engineered probiotics have been designed to treat IBD mainly related to microbiota imbalance (Table 2) [175]. *E. coli* Nissle 1917 (ECN) was genetically engineered to produce a curly fiber matrix that promotes intestinal epithelial integrity and the matrix’s trefoil factor (TFF) component that promotes intestinal barrier function and epithelial repair [176]. The genetic modification of ECN for IBD treatment involved introducing genes to enable the expression of interleukin-10 (IL-10), the ketone body (R)-3-hydroxybutyrate (3HB), and the immunomodulatory protein Sj16 from *Schistosoma*, along with other substances to treat intestinal-related inflammation [174]. These substances can protect the intestinal mucosa by fostering the growth of beneficial probiotic bacteria, suppressing the proliferation of harmful bacteria, enhancing the overall intestinal environment, and reducing the activity of cells or proteins associated with inflammatory responses. This contributes to the treatment and alleviation of symptoms associated with intestinal inflammation associated with IBD [176]. 

Gurbatri et al. utilized synthetic biology techniques and engineered a modified *E. coli* strain named “SLIC” for tumor treatment. This strain colonizes at tumor cells and releases nano-antibodies targeting programmed cell death-ligand 1 (PD-L1) and cytotoxic T lymphocyte-associated protein-4 (CTLA-4) using a stabilized lysing release mechanism. Consequently, this process inhibits tumor growth and prevents metastasis [177]. Similar research has been conducted using engineered EcN to target the angiogenic inhibitor TUM-5 and tumor suppressor p53, as well as converting tumor metabolic waste into L-arginine, which boosts the anti-tumor immune response [178]. Additionally, in their study, Penghao et al. revealed that they developed genetically modified microbes targeting tumors, specifically the transgenic microorganism EcM-GDH (*E. coli* MG1655), which expresses exogenous glucose dehydrogenase (GDH) [179]. These engineered bacterial cells competitively deplete glucose in colorectal tumor cells, triggering pro-death autophagy and p53-initiated apoptosis. This approach serves as a metabolic intervention and starvation therapy. The EcM-GDH cells show a high specificity for tumor tissue, accumulating within it and achieving a favorable tumor/liver distribution ratio, thereby enhancing the selectivity for tumor cells [179]. Yuqing et al. demonstrated the activity of bioengineered *L. reuteri* as a noninvasive delivery method of peptide-based therapeutics such as a Kv1.3 blocker for immunomodulation in rat models of atopic dermatitis and rheumatoid arthritis [180]. In addition, the *L. lactis* strain was engineered to be used for the production of lycopene with a prospected potential of intestinal oxidative damage prevention by decreasing the intracellular ROS level [181]. Other strategies that have the potential to change the human gut microbiome have been tested. For example, antimicrobial peptides (AMPs) produced by genetically modified *E. coli* have been used to target pathogens such as *Pseudomonas aeruginosa* and significantly reduce biofilm formation [182]. 

Researchers have explored the potential of genetically engineered *S.* Typhimurium as a promising avenue for cancer therapy. Modifications have been made to enhance its safety and effectiveness, with a focus on decreasing toxicity and improving selectivity for cancer cells [183]. This bacterium serves as a live delivery vector for various anti-tumor therapeutic agents, including oncolytic viruses and tumor-suppressing proteins [184]. Engineered *Salmonella* serotypes can be made imageable, producing bioluminescence or fluorescence signals to monitor bacterial migration to tumors, aiding in predicting therapeutic efficacy [185]. Tumor-targeting therapies involve the use of *Salmonella* carrying specific RNA, such as short hairpin RNA (shRNA) against inhibin alpha subunit (INHA) for treating late-stage cancers [186]. Furthermore, it has demonstrated potential in combination therapy, either alone or in conjunction with conventional treatments, effectively suppressing metastatic tumor growth [185]. Despite these advancements, challenges persist, necessitating ongoing efforts to address safety concerns and optimize the bacterium’s efficacy in cancer treatment [184]. Ongoing clinical trials are actively exploring the potential of genetically modified probiotics, particularly in the context of treating IBD. Noteworthy examples include a 2006 phase I clinical trial conducted by Braat et al., which assessed the safety and efficacy of a genetically modified *L. lactis* strain [187,188]. Additionally, Kang et al.’s 2018 study delved into the capabilities of engineered probiotics for IBD treatment [174]. These collective efforts underscore the ongoing endeavors to develop and assess the potential of genetically modified probiotics for therapeutic purposes. In conclusion, the genetically modified probiotics have the potential to treat a variety of diseases including IBD, diarrhea, Parkinson’s disease, metabolic diseases, cancer, and bacterial infections such as *H. pylori*, and *C. difficile* [174,188]. Nevertheless, it is crucial to acknowledge that the safety and efficacy of these probiotics remain under investigation.

**Table 2:** Examples of genetically engineered probiotics.

Vehicle	Disease	Mechanism	References
Lactic acid bacteria	Arthritis	Production of anti-inflammatory cytokines like IL-4 and IL-10, which can help suppress TNF-α production and neutrophil influx in the joints and reduce inflammation.	[189]
*B. ovatus* D-6	Cancer	Increases the production of TNF-α-specific IgG and IgM in the body, promoting an immune response against cancer cells.	[190]
*B. longum*	Cancer	Expresses tumstatin or other anti-angiogenic proteins that have the potential for antitumor therapy in tumor-bearing mice by proliferation inhibition and apoptosis induction in vascular endothelial cells.	[189]
*E. coli* Nissle 1917	Cancer	Expresses glucose and ribose sugars receptor Trz1, which, upon activation, triggers the expression of a green fluorescent protein (GFP) reporter within tumors correlated with tumor cell viability. Targets and restricts mouse B16 melanoma and 4T1 breast tumors through the expression of azurin protein.	[191,192]
*S.* Typhimurium	Cancer	*S*. Typhimurium with a modified sidA gene undergoes lysis upon tetracycline exposure, leading to the release of agents causing cell cycle arrest in majority of MCF7 breast cancer cells in the subG1 population.	[193]
*S.* Typhimurium	Cancer	Employs quorum sensing-regulated lysis, causing the discharge of anti-cancer substances within tumors linked to heightened activation of T cells infiltrating tumors, swift regression of tumors, prevention of metastasis, and prolonged survival in preclinical models.	[194]
*S.* Typhimurium	Cancer	Expresses interferon-gamma (IFN-γ) fused to the N-terminal region of *SipB*, allowing for the efficient secretion of IFN-γ from the bacterium and enhancing the localized delivery of IFN-γ for improved melanoma cells cancer treatment outcomes.	[195]
*B. acidifaciens* JCM	Treatment of infection	Modulates host immune responses and enhances the production of gut IgA levels in gnotobiotic mice.	[196]
*B. longum*	Ulcerative colitis	Colonizes in the intestinal gut, expresses bioactive alpha-melanocyte-stimulating hormone (α-MSH) and exhibits a significant anti-inflammatory effect.	[197]
*L. lactis*	Crohn’s disease	Reducing inflammation and mucositis by secretion of cytokines.	[197]
*B. subtilis*	*H. pylori*	Displaying *H. pylori* antigens on its spore coat and eliciting a Th1/Th17-polarized immune response in a murine model showing potential as an oral vaccine candidate for *H. pylori* infection and reduction in stomach bacterial load.	[189,198]
*L. lactis*	Multidrug-resistant *Enterococcus* spp.	Detects *Enterococcus faecalis* pheromone cCF10 and responds by producing and secreting antienterococcal peptides (bacteriocins), which can kill multidrug-resistant *E. faecalis*.	[189]
*Salmonella* sp.	*Salmonella* and Cholera infection	*S.* Typhimurium vaccine strain expressing *Vibrio cholera* toxin antigen subunit-B heterologous antigen (CtxB) can protect against both salmonellosis and cholera infection. The polyvalent vaccine Z234-pMS101, which expresses CtxB antigen, increases mucosal response and pro-inflammatory cytokine production to be efficacious against both salmonellosis and cholera.	[199]
*E. coli* Nissle 1917	Hepatic steatosis	Expressing fructose dehydrogenase (FDH) or mannitol-2-dehydrogenase (mtIK), leading to reduction in lipid peroxidation, an increase in antioxidant enzyme activities, and the restoration of liver injury marker enzymes.	[200]
*L. lactis*	HIV infection	Oral immunization with *L. lactis* expressing *Streptococcus pyogenes* T3 pilus fused to an HIV antigen gag P24 (LL-Gag) induces strong mucosal immunity in the gut displaying 3-fold higher CD8 T cell responses.	[201]
*L. plantarum* NC8	Hypertension	Expressing angiotensin-converting enzyme inhibitory peptide (ACEIP) coding sequences from TFP and YFP joined by an arginine linker increasing the levels of nitric oxide in the plasma, heart, and kidney and reducing the levels of decrease in the levels of endothelin and angiotensin II.	[189]
*L. lactis*	IBD	Production and delivery interleukin-10 (IL-10) using stress-inducible controlled expression system and delivery of IL-10 cDNA cassette into host cells and LL-IL-27 mediated through mucosal delivery, resulting higher expression of anti-inflammatory cytokines.	[202,203,204]
*B. longum*	IBD	Produces and delivers IL-10 in vivo and regulates immune responses offering therapeutic benefits for inflammatory diseases such as inflammatory bowel disease (IBD) and ulcerative colitis.	[197]
*Streptococcus gordonii*	IBD	Recombinant strain of *S. gordonii* produces bioactive human interleukin 1 receptor antagonist (IL-1ra) through RVFP/IL-RA in vitro and could be suitable for selective targeting of the mucosal surface as a delivery system for inflammatory diseases such as IBD.	[205]
*B. ovatus* V975	Intestinal inflammation	Reduction in inflammatory infiltrate and mucin depletion in the colon, as well as a decrease in epithelial erosion symptoms of DSS-induced colitis in mice.	[190]
*E. coli* Nissle 1917	Obesity	EcN expressing acylphosphatidylethanolamines (NAPEs) or GLP-1 analog may have potential as a therapeutic intervention for obesity, leading to the inhibition of weight gain, food intake, adiposity, insulin resistance, and hepatosteatosis reduction and maintaining lower plasma leptin and insulin levels.	[206]
*L. lactis* SAGX0085	Oral and intestinal mucositis	Secretes human trefoil factor 1 (hTFF1), which is believed to promote cell differentiation and limit cell proliferation and apoptosis to potentially improve the repair of oral and intestinal epithelial damage.	[207]
*L. gasseri*	Type 1 diabetes	Expressing GLP-1(1-37) has been shown to reprogram intestinal epithelial cells into insulin-secreting cells in rats, leading to a reduction in blood glucose levels. In a study, diabetic rats fed with *L. gasseri* expressing GLP-1(1-37) had significantly higher insulin levels and were more glucose-tolerant than those fed with wild-type *L. gasseri*.	[208]
*L. lactis*	Type 1 diabetes	Secretes human pro-insulin and induces antigen-specific immune tolerance in T1D by delivering cytokines like IL-10 and IL-4 that can restore the tolerance of pancreatic beta cells.	[9]

## 9. Conclusions and Future Prospective

Research on NGPs holds a great promise for developing pharmaceutical treatments, and it is a rapidly evolving field. The swift progress in advanced genetic sequencing tools, bioinformatics platforms, and powerful tools for editing bacterial genomes has sparked increased interest in exploring new probiotic strains for biomedical applications. NGPs exhibit the ability to target specific diseases and function therapeutically similar to drugs. Particularly, they show promise in addressing chronic inflammation-related conditions like colitis, IBD, IBS, obesity/metabolic syndromes, diabetes mellitus, liver diseases, cardiovascular diseases, cancer, and neurodegenerative diseases. However, further research is necessary to fully grasp the potential of NGPs, ensuring a comprehensive understanding of their safety and efficacy in human applications.

The future development of NGPs is marked by innovative strategies aimed at revolutionizing probiotic therapies. One pivotal direction involves the pursuit of personalized probiotics, acknowledging the highly individualized nature of the human gut microbiome. Through techniques like metagenomic sequencing, researchers are striving to identify specific bacterial strains that offer maximum benefits to individual patients. This personalized approach tailors probiotic therapies to meet the unique needs of each person, promising more effective and precisely targeted interventions. Another crucial avenue is the integration of synthetic biology and gene editing techniques to enhance NGPs. This entails developing probiotics with advanced properties, including improved survival in the gut, precise targeting within specific regions of the gastrointestinal tract, and the capability to produce therapeutic compounds. Concurrently, researchers are exploring the synergy of probiotics with other therapeutic modalities, such as prebiotics, postbiotics, or traditional drugs, to unlock synergistic effects. Additionally, the evolution of sophisticated delivery systems, like nanoparticles and time-release formulations, is underway to ensure targeted delivery to specific gut regions or cell types, sustaining exposure to beneficial bacteria. The overarching goal is to harness these advancements for the development of NGPs that address individual health conditions, ranging from IBD to allergies and metabolic disorders, ushering in a new era of precision medicine in the realm of gut health. Taken together, the future of NGPs involves a shift toward personalized, enhanced, and targeted probiotic therapies, exploring combinations with other treatments and utilizing advanced technologies for delivery and development.

## Figures and Tables

**Table 1:** Comparison between next-generation probiotics and traditional probiotics.

	Next Generation Probiotics	Traditional Probiotics
Origin	Derived from next-generation microorganisms that have been recently isolated using advanced tools and techniques	Long history of use and are derived from a limited number of species, such as *Lactobacillus* and *Bifidobacterium*
Development	Developed based on comparative analysis of microbiota compositions between healthy and diseased individuals	Developed through a top-down screening strategy, which involves screening microbes enriched in healthy individuals compared to those in diseased individuals
Safety	Their safety is not yet proven as they are relatively new and have not been used for as long as traditional probiotics	A long history of safe use in humans
Applications	Primarily used to treat or cure disease conditions	Mainly used as food ingredients or supplements.
Regulation	Considered to be live biotherapeutic products (LBPs) or drugs, which are subject to pharmaceutical clinical trials and research on their pharmacokinetics and pharmacodynamics	They are not subject to the same level of regulation
Strain specificity	Their health-promoting features are more closely tied to specific strains rather than entire species	They are species-specific

## Data Availability

Not applicable.
